# Can we be certain about future land use change in Europe? A multi-scenario, integrated-assessment analysis

**DOI:** 10.1016/j.agsy.2016.12.001

**Published:** 2017-02

**Authors:** I.P. Holman, C Brown, V Janes, D Sandars

**Affiliations:** a School of Water, Energy and Environment, Cranfield University, Cranfield, Bedford MK43 0AL, UK; b School of Geosciences, University of Edinburgh, Edinburgh EH8 9XP, UK

**Keywords:** Climate change, Socio-economic change, Impacts, Integrated assessment, Uncertainty

## Abstract

The global land system is facing unprecedented pressures from growing human populations and climatic change. Understanding the effects these pressures may have is necessary to designing land management strategies that ensure food security, ecosystem service provision and successful climate mitigation and adaptation. However, the number of complex, interacting effects involved makes any complete understanding very difficult to achieve. Nevertheless, the recent development of integrated modelling frameworks allows for the exploration of the co-development of human and natural systems under scenarios of global change, potentially illuminating the main drivers and processes in future land system change. Here, we use one such integrated modelling framework (the CLIMSAVE Integrated Assessment Platform) to investigate the range of projected outcomes in the European land system across climatic and socio-economic scenarios for the 2050s. We find substantial consistency in locations and types of change even under the most divergent conditions, with results suggesting that climate change alone will lead to a contraction in the agricultural and forest area within Europe, particularly in southern Europe. This is partly offset by the introduction of socioeconomic changes that change both the demand for agricultural production, through changing food demand and net imports, and the efficiency of agricultural production. Simulated extensification and abandonment in the Mediterranean region is driven by future decreases in the relative profitability of the agricultural sector in southern Europe, owing to decreased productivity as a consequence of increased heat and drought stress and reduced irrigation water availability. The very low likelihood (< 33% probability) that current land use proportions in many parts of Europe will remain unchanged suggests that future policy should seek to promote and support the multifunctional role of agriculture and forests in different European regions, rather than focusing on increased productivity as a route to agricultural and forestry viability.

## Highlights

• Future European land systems modelled across scenario space.
• Substantial consistency in locations and types of change even under divergent conditions
• Currently marginal agricultural areas may face complex and difficult choices.

## 1 Introduction

Humans have been changing the European landscape for millennia in response to their requirements for the many benefits or ecosystem services arising from the natural environment and its constituent resources. Developments in social systems, new technologies and crops, growing populations and economies have all had dramatic effects (Antrop, 2005, Fischer-Kowalski and Haberl, 2007). Climatic changes have also had substantial impacts, both on the landscape and on human societies, driving a complex pattern of inter-related environmental changes (Messerlia et al., 2000, Büntgen et al., 2011). Now, as the pace of socio-economic and climatic change continues to quicken, their consequences for the land system are commensurately greater and more uncertain. Climate change is likely to have impacts through changes in precipitation, temperature, CO_2_ concentrations and sea level rise, affecting the suitability of land for different crops (Iglesias et al., 2012, Bindi and Olesen, 2011), tree species (Hanewinkel et al., 2013), habitats (e.g. Lehsten et al., 2015) and forms of management (e.g. irrigation - Garrote et al., 2015). Meanwhile, human activities will further modify the European landscape across scales, as populations, dietary preferences, trading patterns and management practices all change (Holman et al., 2008, Holman et al., 2016, Harrison et al., 2013, Rounsevell et al., 2006).

Previous research suggests that these non-climatic pressures may be more important drivers of land use change than climate change (Holman et al., 2005, Rounsevell and Reay, 2009). However, the diversity of social, economic, political and technical factors involved mean that future demands for living space and natural resources are hard to predict. This is exemplified by the breadth of potential socio-economic storylines used in impact assessments, from the global-to-regional Special Report on Emissions Scenarios (SRES; Nakicenovic and Swart, 2000) and Shared Socioeconomic Pathways (SSP; O'Neil et al., 2014) to the continental-to-national storylines developed by stakeholders (e.g. Kok et al., 2015, Metzger et al., 2010). Further uncertainty arises from differences between methods of analysis and modelling that emphasise distinct processes or sectors. These often give divergent projections even under identical climatic and socio-economic scenario conditions, suggesting that the identification and representation of major land change drivers requires significant improvement. In particular, the discrete sectoral nature of many models precludes consideration of the numerous cross-sectoral interactions that influence land use distributions (Harrison et al., 2016). Examples include changes in urban extent or coastal flood defence policy affecting agricultural land availability (Mokrech et al., 2008), and changes in population and water consumption affecting availability of water for irrigation (Henriques et al., 2008). Omitting such interactions can lead to substantial over- or under-estimation of climate impacts and direct and indirect consequences for land use (e.g. Reidsma et al., 2006, de Moel and Aerts, 2011, Di Lucia et al., 2012, Harrison et al., 2016).

One important outcome of uncertainty-focused model applications has been the identification of areas where high levels of uncertainty imply that land use is especially vulnerable to change. However, far less has been discovered about the areas where land use is robust to climatic and socio-economic pressures, and therefore the conditions which allow for the maintenance of food supplies, livelihoods, biodiversity and other ecosystem services. For this purpose, comprehensive scenarios and integrated modelling frameworks are particularly valuable because they allow for fuller, more realistic representations of the land system. Previous examples include studies focusing on future crop yields (Ewert et al., 2015, Wiebe et al., 2015), agricultural land use change (Piorra et al., 2009), broader land and/or energy usage (Verburg et al., 2008, Stürck et al., 2015, van Vuuren et al., 2016), inter-sectoral climate impacts (Fischer et al., 2005, Frieler et al., 2015), and policies for the sustainable development of land use systems (van Delden et al., 2010, Reidsma et al., 2011). Many of these studies involve integrated modelling of European land use; a particularly interesting case due to the research attention it has received in support of a coherent political system that attempts to influence land use outcomes across scales.

While Europe therefore provides a setting where advances in understanding of future land use change should be both possible and of practical value, this potential has not yet been fully realised. In particular, there has been a lack of assessment of the extent of certainty across land use categories under climatic and socio-economic changes acting directly and indirectly through representative cross-sectoral interactions. This paper addresses this gap using an integrated multi-sectoral modelling platform, the CLIMSAVE Integrated Assessment Platform (IAP), which incorporates a broader range of drivers and cross-sectoral processes than previous integrated models, and so allows for less strongly conditional projections of future land use change. We address two research questions:

1. Can highly uncertain futures lead to certain outcomes for European land use in the 2050s?
2. Where in Europe are the current distributions of land uses unlikely to change significantly in the medium term?

We use findings from the IAP to examine the conditions that generate projected stability, and their implications for political interventions intended to maintain land system functionality under global change.

## 2 Methods

### 2.1 The CLIMSAVE IA Platform

**Fig. 1:**
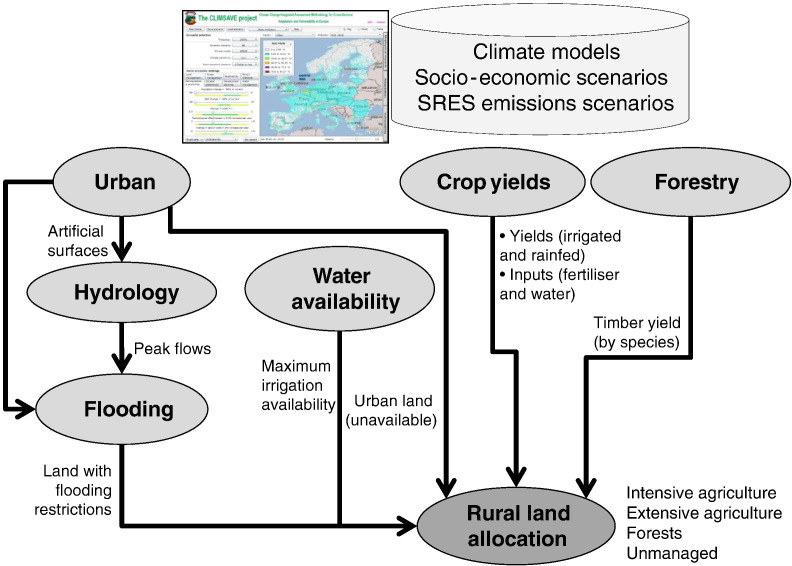
Schematic of the model system interlinkages determining rural land allocation within the CLIMSAVE IAP.

The CLIMSAVE^1^ IA Platform (IAP) is an interactive, exploratory, web-based tool for simulating climate change impacts and vulnerabilities on a range of sectors (Harrison et al., 2013, Harrison et al., 2015a). The Platform integrates a suite of models of urban development, water resources (Wimmer et al., 2015), coasts (Mokrech et al., 2015), agriculture and forests (Audsley et al., 2015), and biodiversity (Dunford et al., 2015a) to simulate the spatial effects of different climatic and socio-economic scenarios across Europe (Fig. 1). The IAP has been applied widely in climate change impact (Audsley et al., 2015, Holman et al., 2016, Wimmer et al., 2015, Harrison et al., 2015b, Mokrech et al., 2015, Harrison et al., 2016), adaptation (Dunford et al., 2015a) and vulnerability (Dunford et al., 2015b) assessments, in robust policy analysis (Jäger et al., 2015) and has been tested extensively through model sensitivity (Kebede et al., 2015) and uncertainty analyses (Dunford et al., 2014, Brown et al., 2015). The Platform operates at a spatial resolution of 10 arcmin × 10 arcmin (approximately 16 km × 16 km in Europe) grid cells, although multiple soil types are represented in each grid cell, and covers two thirty-year timeslices (2020s and 2050s).

### 2.2 Climate and socio-economic scenarios

#### 2.2.1 Climate scenarios

The climate change scenarios within the IA Platform are based on combinations of the IPCC emissions scenarios (A1b, A2, B1 or B2), three climate sensitivities (low, medium or high) and five global climate models (GCMs). The five GCMs (MPEH5, CSMK3, HadGEM, GFCM21 and IPCM4) were chosen from the CMIP3 database using an objective method to represent as much uncertainty as possible due to between-GCM differences (see Dubrovsky et al., 2015 for further details). Projections of Europe-wide area-average temperature change across these climate models and scenarios range from 1.1 to 4.9 °C in winter and from 1.0 to 3.6 °C in summer in the 2050s. Projections for precipitation change range from increases of between 1.1 and 12.5% in winter and decreases of between 2.0 and 29.5% in summer. The pattern of temperature and precipitation changes differs according to the GCM (see Online Resource 2 of Harrison et al., 2015b). Although we acknowledge that there are more recent scenarios available than those in the IAP, the European area-average changes across these scenarios cover at least the 25th to 75th percentile range of the European changes in summer and winter precipitation and temperature change to 2065 for the CMIP5 global models for the RCP2.6 to RCP8.5 scenarios (Christensen et al., 2013).

#### 2.2.2 Socio-economic scenarios

The IAP contains four European socio-economic scenarios that were developed by stakeholders in a series of professionally-facilitated participatory workshops (see Gramberger et al., 2015). In the first and second workshops, the objectively selected stakeholder group developed and iterated qualitative socio-economic stories and dynamics according to the two drivers that they considered most important and uncertain: “[*effective* vs *ineffective*] solutions by innovation” and “[*gradual* vs *roller-coaster*] economic development”. This produced four scenarios which describe the contrasting evolution of a range of social, economic, cultural, institutional and political drivers in Europe (Kok et al., 2015):

• We Are the World – effective governments change the focus from GDP to welfare, which leads to a redistribution of wealth, and thus to less inequality and more (global) cooperation;
• Should I Stay or Should I Go – a failure to address economic crises leads to an increased gap between rich and poor, political instability and conflicts;
• Icarus – short-term policy planning and a stagnating economy lead to disintegration of social fabric and the shortage of goods and services;
• Riders on the Storm – strong economic recessions hit hard, but are successfully countered with renewables and green technologies. Europe is an important player in a turbulent world.

The qualitative stories and quantitative models were linked in a transparent and reproducible way using a “fuzzy set theory methodology” (Kok et al., 2015) in the first and (refined in) the second workshops to determine scenario-specific quantitative values (Supplementary Material Table SM1), expressed relative to the value in the baseline (2010) socioeconomics. These were then used to inform expert-determined values of the remaining model inputs that were representative of the stakeholders' stories.

### 2.3 Models

A meta-modelling approach based on computationally efficient or reduced-form models that emulate the performance of more complex models (Harrison et al., 2013) was used to facilitate greater complexity of model linkages within the IA Platform and a relatively fast run time. Although the CLIMSAVE IAP includes a large number of interlinked models (Fig. 1), this section briefly describes those models which indirectly or directly affect spatial land allocation. For further details on the models, see Harrison et al., 2015a, Harrison et al., 2016 and papers cited.

• Urban expansion: The Regional Urban Growth (RUG) metamodel consists of a look-up table of percentage of artificial surfaces per grid cell (between zero and almost 100%) derived from running the RUG model (based on Reginster and Rounsevell, 2006) with all possible combinations of input values (population, GDP, household preference for proximity to green space versus social amenities, attractiveness of the coast (scenic value versus flood risk) and strictness of the planning regulations to limit sprawl). Development in urban and rural areas is given first priority in the allocation of land;
• Flooding: The Coastal Fluvial Flood (CFFlood) meta-model (Mokrech et al., 2015) is a simplified process-based model that identifies the area at risk of flooding based on topography, relative sea-level rise or change in peak river flow and the estimated Standard of Protection of flood defences. The probability of flood inundation constrains the allocation of land for agriculture, with land with a > 10% and > 50% annual probability of flooding being unsuitable for intensive agriculture and extensive agriculture, respectively, according to Mokrech et al. (2008).
• Water: The WaterGAP (WGMM) meta-model (Wimmer et al., 2015) uses 3D response surfaces to reproduce WaterGAP3 runs at a 5′ × 5′ resolution for about 100 spatial units (single large river basins or clusters of smaller, neighbouring river basins with similar hydro-geographic properties). The difference between simulated water availability and projected non-agricultural water consumption determines the maximum water available for agricultural irrigation in each spatial unit;
• Forest: MetaGOTILWA + (Audsley et al., 2015) is an artificial neural network (ANN) that emulates GOTILWA + (Gracia et al., 1999). The ANN was trained on GOTILWA results for 889 grid cells across Europe, and simulates average timber yields for a range of deciduous and coniferous tree species under different management regimes and soil characteristics;
• Crops: The crop yield metamodels (Audsley et al., 2015) use ANNs to predict the average yield of a range of annual and permanent crops under rainfed and irrigated conditions. They have each been trained and validated on simulated outputs across Europe from the daily ROIMPEL model (Audsley et al., 2006) for winter and spring wheat, barley and oilseed rape, potatoes, maize, sunflower, soya, cotton, grass and olives. The training datasets were sampled from 150,000 model data points to adequately cover the range of soil (Panagos et al., 2012) and climate predictors and the predictands.
• Rural land allocation: The SFARMOD meta-model (Audsley et al., 2015) allocates available land across Europe based on profit and other constraints (urban land use, irrigation availability; food and timber demand). It uses a series of regression equations to simulate the behaviour of the full SFARMOD-LP model, a mechanistic farm-based optimising linear programming model of long-term strategic land use. The metamodel was fitted to SFARMOD-LP outputs from 20,000 randomly selected sets of input data that fully cover the current and future parameter input space. The regression is broken into steps that estimate first the percentage of the area of each crop in each grid cell, then the costs of dairy cows (concentrates), then the fixed costs of labour and machinery, from which gross margins, net income and profit is derived. Up to 10 iterations adjust crop and livestock prices to meet the demand for food within Europe, which is a function of population, imports, food preferences and bioenergy. Where the resulting profit is above a threshold (set at €350/ha) land is deemed to be used for intensive agriculture (either arable or dairy agriculture). Otherwise the profit is re-calculated without the arable crops to represent extensive agriculture (sheep and beef) and compared with the profit from managed forests (based on the annual equivalent profit of a total Net Present Value over the life of the forest). If the resulting profit is greater than a second threshold (set at €150/ha) then this land is used for whichever of managed forest or extensive agriculture has the greatest profit. Otherwise the land is not used for productive purposes – it is assumed to be unmanaged forest if the Net Primary Productivity of unmanaged forests is positive and greater than the grass yield of extensive grass, else unmanaged land.

All metamodels were satisfactorily validated against either baseline observations or the validated outputs of the full model (see Holman and Harrison, 2011 for the validation of each model; and the sensitivity analysis of the linked models within the IA Platform in Kebede et al., 2015).

### 2.4 Model runs and analysis

The CLIMSAVE IA Platform was run for 300 scenarios for the 2050s timeslice to explore the effects of climate change and socio-economic change uncertainties on European land use change. The scenario combinations can be categorised into two scenario groups:

• *Climate change only* - Climate scenarios for every combination of the 4 emissions scenarios, three levels of climate sensitivity and five GCMs, combined with baseline socio-economics (60 runs);
• C*limate and socio-economic change* - Climate scenarios (60 runs above) combined with each of the four socio-economic scenarios (240 runs).

Four indicators from the IAP were analysed: 1) area of intensive agriculture; 2) area of extensive agriculture; 3) forest area; and 4) area of unmanaged land. In all analyses, it was assumed that each scenario was equally probable i.e. no prior assumptions were made regarding the likelihood of a particular magnitude of climate or socio-economic change. Grid based results for each of the 60 and 240 simulations were averaged to provide maps of the multi-scenario means for climate change only and climate and socio-economic change simulations in the 2050s, which were compared against the simulated baseline distribution to assess whether there is spatial coherency within the two scenario groups despite the diverse climate and socio-economic scenarios.

Secondly, we investigated whether there are areas in Europe in which individual land uses have future spatial certainty in the direction of change in their spatial extent. There is no common definition of what constitutes important land use change, with recent land cover change across Europe ranging from < 0.1% per annum (as a percentage of national area) in Norway, Malta, Poland and Slovenia to > 0.5% per annum in Portugal and Slovakia (European Environment Agency, 2010) and scenario studies projecting changes in the spatial extent of individual European land use classes of up to 5% by 2020 (e.g. European Centre for Nature Conservation, 2006) and 8% by 2050 (e.g. Rounsevell et al., 2005) compared to 2000. We adopted a threshold of 5%, but tested the robustness of results across a range of thresholds from 0.1% to 25%. The probability of changes in extent of each land use within each grid cell of > 5% of the simulated baseline (using 1961–90 climate and 2010 socio-economics) was calculated and classified according to the likelihood scale of Mastrandrea et al. (2010) to identify those cells in which an increase or decrease in land use extent is “likely” (66–90% probability) or “very likely” (> 90% probability). Cells in which no change in the spatial extent of each individual land use greater than ± 5% was “very likely” were also identified. Finally, the probability that none of the four land uses change in extent by > 5% was calculated for each grid square to identify areas where there is spatial certainty that all land uses will remain largely unchanged in extent. These analyses were then repeated across the range of thresholds above.

## 3 Results

### 3.1 Multi-scenario average spatial land use allocation

**Fig. 2:**
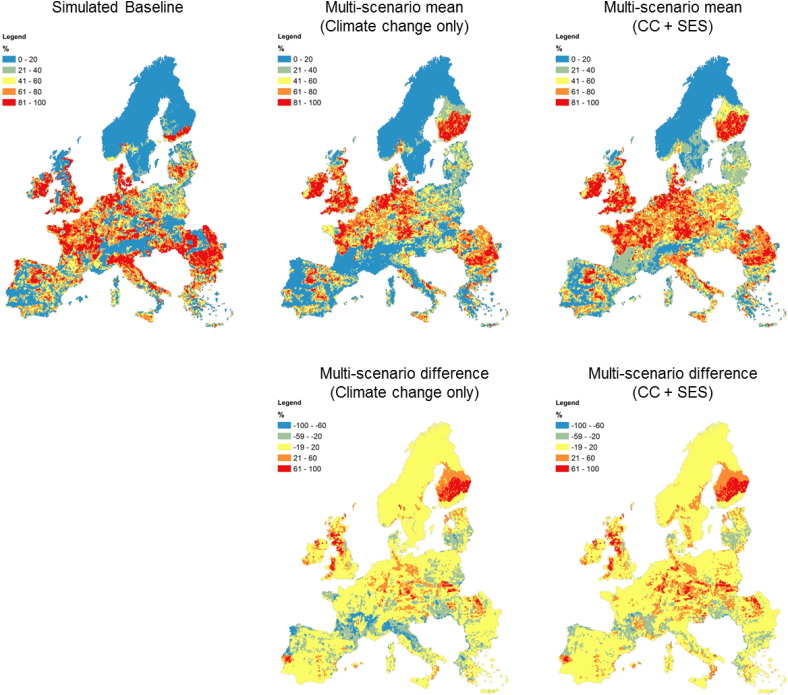
Comparison of the simulated baseline distribution of intensive agricultural land with the multi-scenario mean of the [60] simulations with climate change only and the [240] simulations with climate and socio-economic change for the 2050s.

Given the divergent nature of the socioeconomic scenarios and differing spatial patterns of temperature and precipitation change from the climate models, the simulated baseline distribution of each land use was compared with the multi-scenario mean of the [60] simulations with climate change only and the [240] simulations with climate and socio-economic change (Fig. 2 and Figs. SM2–4). It is apparent that there is a spatial coherency in the simulated future distribution across Europe for each land use; i.e. averaging the individual grid-level values resulting from the diverse range of input scenarios has not led to a quasi-random distribution of land use allocation. Focusing on intensive agriculture, the effect of climate change alone (with baseline economics meaning that there is no change in the food demand or imports) leads to a projected northward migration of intensive agriculture (Fig. 2), particularly into northern UK and Finland. This is also associated with reduced intensive agricultural areas in southern Europe (especially Spain and Italy) due to a combination of increased heat stress and reduced availability of irrigation water. These two factors reduce the simulated relative competitiveness of Mediterranean agriculture in contributing towards meeting the demand for European agricultural production, so that the required demand for production can be met by a smaller agricultural area focused in a band across central and north-western Europe due to yield increases.

The introduction of socioeconomic change affects modelled European agricultural land use requirements by changing both the demand for agricultural production, through changing food demand (due to changes in population, wealth and dietary preferences) and net imports (arising from changes in Europe's relationship with the rest of the world), and the efficiency of agricultural production (through scenario changes in mechanisation, yield development and crop breeding and irrigation efficiency). The multi-scenario mean in Fig. 2 shows the most intensive areas of agriculture (red areas) in the same locations as the climate-only multi-scenario mean (as these are the most profitable production areas) but expands the production area into regions that had lost competitiveness under climate change such as southern France and the Baltic states.

### 3.2 Model certainty in the direction of land use change across Europe

**Fig. 3:**
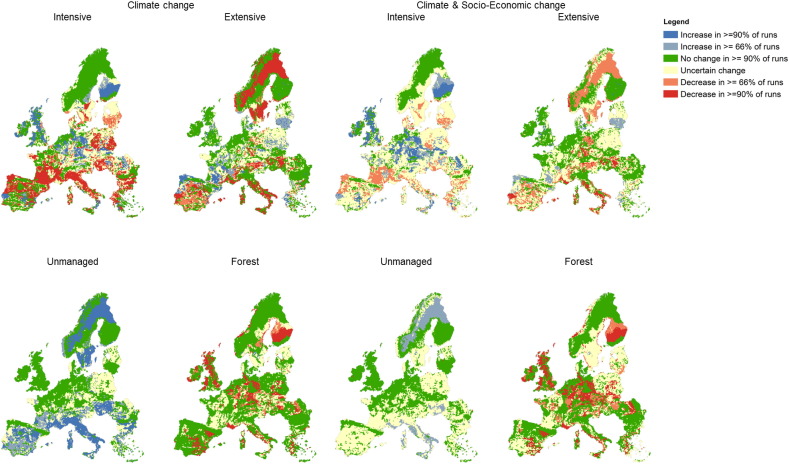
Multi-scenario certainty in the direction of modelled land use change (of at least 5% within a grid cell) for the 2050s for (left) climate change only and (right) climate and socioeconomic change.

Fig. 3 and Fig. SM5 shows the certainty in the modelled direction of change in the spatial extent of each land use class across all of the scenario combinations, expressed as the percentage of runs in which the land use class changes in extent by > 5% (compared to the baseline simulation) in a given grid cell. The sensitivity analysis using change thresholds of between 0.1 and 25% within each grid cell (Fig. SM1 in the Supplementary material) shows that increasing the change threshold inevitably leads to an increasing spatial extent in the area with certain “no change” and decreasing extent of areas with both uncertain change and certain (increasing and decreasing) change. However, the overall percentage in each certainty class is not fundamentally changed, demonstrating the robustness of the results.

With climate change alone, there are areas with significant confidence in the direction of change for all land uses, with forest area decreasing in > 90% of simulations in significant areas of all regions of Europe; unmanaged land increasing in southern Europe and Scandinavia at the expense of intensive and extensive agricultural land; extensive agricultural land increasing through a band across the centre of Europe at the expense of intensive agricultural land; and intensive agriculture increasing in parts of northern Europe at the expense of forest. There are also areas with confidence of no change – for example, intensive agriculture is unchanged within much of central England, northern France and the Benelux countries; whilst forest is little changed in large areas of Scandinavia, Spain and central Europe.

However, there is a major decrease in the confidence of the direction of change and a large expansion of the areas with uncertain change (i.e. < 66% agreement) when the socio-economic scenarios are introduced (Fig. 3 and Fig. SM5). In particular, there are no significant areas with high (> 90% of simulations) confidence in the decrease of intensive agricultural land or increase in unmanaged land. There are only areas within Scandinavia, Italy, France and Hungary in which there is at least a 66% agreement of an increase in unmanaged land. The areas with certainty of no change (change of < 5%) decrease slightly for most land covers.

### 3.3 Model certainty in stable land use patterns across Europe

**Fig. 4:**
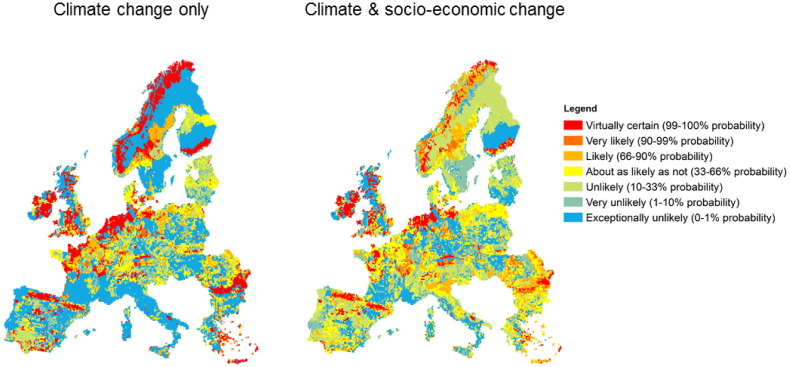
Certainty of simulated baseline extent of land covers remaining unchanged (± 5%) under simulations with climate change only [60] and simulations with climate and socio-economic change [240] in the 2050s.

Fig. 3 showed that there are areas across Europe in which there are high levels of certainty in the direction of change (either increases or decreases) in the extent of individual land use classes due to climate and socioeconomic change. However, it also showed extensive areas across Europe with either certainty of little change or uncertainty in the magnitude or direction of change. Fig. 4 therefore shows the percentage of simulations in which all four land use classes within a given grid cell change by less than ± 5% from the baseline proportions, classed according to the likelihood scale of Mastrandrea et al. (2010). Approximately 20% of the simulated cells across Europe are very likely (90–99% probability) or virtually certain (99–100% probability) to maintain their baseline land use proportions despite the effects of climate change on land suitability and crop and timber yields (Table 1). These stable cells include significant areas within the UK, northern France, northern Spain, Germany and Scandinavia, and account for > 50% of the areas of three countries: The Netherlands (88%), Republic of Ireland (54%) and Norway (51%).

The introduction of the socio-economic scenarios decreases the extent of areas very likely or virtually certain to retain baseline land use proportions from 20% to about 12% of the simulated grid cells (Table 1). Only the Netherlands (54%) and Republic of Ireland (50%) now have > 50% of grid cells in this category. At a national scale, the socio-economic scenarios lead to a reduction in areas of certainty in all countries, with the exception of the Czech Republic and Austria where there is an increase of 1% and 5%, respectively. There are also smaller areas where the certainty of maintaining the current land use distribution increases; for example eastern England, northern Romania and the Po valley in Italy. However, whilst socio-economic scenarios reduce overall land use certainty in Europe, the percentage of cells in which baseline land use proportions are exceptionally unlikely (0–1% probability) to remain unchanged also decreases, from 42% to 20%, with associated increases in cells that are very unlikely (1–10% probability) or unlikely (10–33% probability) to remain unchanged.

## 4 Discussion

Changes in global land systems in the coming decades will be strongly influenced by a range of interacting climatic and socio-economic factors. This complexity makes projections of future change hard to achieve, and reliant on integrated modelling approaches that respect the dynamics that occur within and between individual sectors. As a result, assessments of future conditions, made largely in the absence of well-developed integrated approaches of this kind, have so far focused on areas of uncertainty. However, recent methodological advances allow for more confident exploration of the converse; areas that, with some level of certainty, appear robust to external drivers of change and internal complexity. Here, we applied a cross-sectoral European modelling platform spanning a wide range of climatic and socio-economic conditions, which therefore covers or exceeds the uncertainty space identified in previous studies of future land use change (e.g. Harrison et al., 2016, Brown et al., 2015, Prestele et al., 2016, Verburg et al., 2013, Stürck et al., 2015). Uncertainty in future climate conditions was addressed through sixty climate change scenarios that spanned at least the 25th to 75th percentile range of the European changes in summer and winter precipitation and temperature change to 2065 for the CMIP5 global models for the RCP2.6 to RCP8.5 scenarios, and included differing spatial patterns of change. Furthermore the four socio-economic scenarios included contrasting directions of change in key scenario inputs such as population, GDP, spatial planning policy, societal behaviour (including dietary preferences for meat and water consumption), crop breeding and agronomic improvement that influence land-take for development, food demand, irrigation water availability and agricultural productivity.

Given the extent of uncertainty space investigated here, an entirely divergent set of projected future conditions might be expected. However, we instead find substantial consistencies across results. Indeed, the strong spatial patterns within the multi-scenario means of individual land use distributions in the 2050s (Fig. 2 and Figs. SM2–4) suggest a considerable degree of predictability in future land use at the European scale. In particular, the analysis presented here suggests that there are areas of Europe with high levels of certainty in two aspects of future land use change – the direction of change for individual land uses and the probability of maintaining the current land use distribution.

These findings are consistent with Brown et al. (2015) who found significant overlap in the probability density functions of the grid-based indicators of food production per capita, land use intensity index and land use diversity index for the four contrasting CLIMSAVE scenarios. This consistency is especially notable because the analysis of Brown et al. (2015) included model uncertainty that we do not directly measure here; a potentially large source of uncertainty (e.g. Alexander et al., 2016). Furthermore, some of our broad and specific findings are consistent with those of other integrated and stand-alone models. For example, the magnitudes of change in the agricultural sector are in line with the (albeit wide) range found by the inter-model comparison of Alexander et al. (2016), while our finding that some areas of Europe appear to be robust to a wide range of scenario conditions also agrees with the studies of Verburg et al. (2010), Verburg et al. (2013), Stürck et al. (2015) and Prestele et al. (2016). These earlier studies found substantial areas of stability and/or predictability using more narrowly-based or discrete modelling approaches at European and global scales (which included the effects, for instance, of GDP and population change). These earlier findings also have some similar geographical characteristics, particularly in the locations of agricultural intensification in northern and western Europe and agricultural extensification, diversification or abandonment in parts of southern and eastern Europe (see e.g. Stürck et al., 2015, Fig. 8). These suggest that error propagation in the IAP's cross-sectoral models is not amplifying the impact of individual, sub-model uncertainty, supporting the conclusions of Kebede et al. (2015) and Dunford et al. (2014). The present study represents a valuable advance, therefore, because it encompasses a greater number of socio-economic drivers of change and of sectoral and cross-sectoral processes (also known to be large potential sources of uncertainty; Harrison et al., 2016), thereby allowing for the identification of particular characteristics that lead to certainty in land use futures.

Some of the most informative and novel findings presented here relate to the ways in which specific drivers affect, or fail to affect, land use patterns. For instance, we found that climate change was associated with a number of key simulated changes. A decrease in forest area was likely (66–100% probability) in many regions of Europe due to large increases in tree growth simulated by metaGOTILWA + under climate change. This suggests a direction of change and degree of certainty not identified by previous studies (e.g. Stürck et al., 2015), which may result from differences in underlying assumptions about climate impacts on tree growth and socio-economic impacts on wood demand (Eggers et al., 2008). In our simulations, climate change also drove agricultural changes (Fig. 2) that appear to reinforce the current trends of intensification of agriculture in northern and western Europe and extensification and abandonment in the Mediterranean region (Levers et al., 2015), as shown by the likely (66–100% probability) decreases in intensive and extensive agriculture and likely increase in unmanaged or very low intensity agriculture in many parts of southern Europe (Fig. 3). The combination of increased heat stress due to higher temperatures, increased summer drought stress in rainfed systems and reduced availability of irrigation water in the region all contribute to reducing profitability and competitiveness of Mediterranean agriculture compared to that in central and northwestern Europe. This suggests that established findings about the impacts of climate change on European agriculture (e.g. Olesen and Bindi, 2002, Bindi and Olesen, 2011) are largely robust to socio-economic change and cross-sectoral interactions, as also indicated by earlier integrated modelling studies (e.g. Verburg et al., 2013, Stürck et al., 2015).

We also find that socio-economic drivers of land use change play a crucial role, sometimes even dominating over climatic drivers. For instance, the introduction of socio-economic scenarios partly offsets the loss of agricultural land in southern Europe leading to a (small) increased likelihood of stable land systems in very unstable areas (as shown by grids shifting from “exceptionally unlikely” to remain unchanged to “unlikely or “very unlikely” – Table 1). This arises as large increases in European food demand in some scenarios (due to population growth, increased affluence and reduced net imports) allow more marginal areas to continue to contribute profitably to production. This is also true of areas such as the Po valley in Italy with its deep water-retentive soils. Nevertheless, the total proportion of grid cells which are very likely (90–99% probability) or virtually certain (99–100% probability) to maintain baseline land use proportions decrease from 20% of grid cells under climate change alone to just 12% with both climate and socio-economic change as a consequence of the major differences in food and timber demand, agricultural productivity and food trade across the divergent socio-economic scenarios, which together determine the requirements for land . These stable areas are mostly in north-western Europe and Scandinavia, while many parts of the Mediterranean have a high likelihood of changing land use distributions (with the main exception of the Pyrenees and Cantabrian mountain chains in northern and north-eastern Spain respectively). In fact, extensification or abandonment in currently marginal areas, as agronomic conditions become less favourable through increased summer drought stress, reduced availability of irrigation water and decreasing profitability, give a high likelihood of land use change in much of Spain, southern France and Italy (Fig. 3). Elsewhere (e.g. Scandinavia), climatically-driven increases in timber yields act alongside increasing demands for agricultural land to substantially reduce the projected area of forestry, especially where timber prices also fall (Brown et al., 2015).

It is apparent from these results that some specific factors promote or impede land use change in general. Particularly influential were population growth rates, dietary preferences, yields and imports (Kebede et al., 2015, Audsley et al., 2015), with each having direct consequences for the extent of agricultural land required. Areas that are robust to variations in these factors, and therefore relatively certain of maintaining current land uses, have a number of key characteristics – (1) mountainous areas in which topography (slope) and soil depth constrain land use options, such as the Pyrenees and the Pico de Europa in Spain; (2) relatively wet and mild areas with maritime climate influences which support strong grass growth but remain unsuitable for conversion to arable agriculture, such as parts of the Republic of Ireland and central England; and (3) intensive agricultural areas with deep, water-retentive soils and favourable climates that assure continuing profitability and competitiveness, such as the Netherlands. In contrast, autonomous adaptation within much of southern Europe is likely to be insufficient to offset reduced relative profitability compared to northern and western Europe, where competitiveness increases through increases in yield and expansion of climatically suitable areas. Therefore, further adaptation strategies (autonomous or planned) in the Mediterranean and south-eastern parts of Europe should not focus on trying to increase productivity as a route to agricultural profitability but rather strike a balance between the economic, environmental and social functions of different European regions that recognises the multifunctionality of land use (Bindi and Olesen, 2011, Brown et al., 2016a).

While our results are valid across a large area of uncertainty space due to our use of several integrated models and socio-economic scenarios, there is some residual potential for uncertainty. In particular, model uncertainty (in terms of the design and form of integration of models) is known to be an important source of uncertainty in land change projections (Alexander et al., 2016; Dormann et al., 2007, Olesen et al., 2007, Harrison et al., 2016), and one that we do not consider beyond the use of several distinct GCMs in our simulations. This may be particularly significant where the decision-making of land managers and policy-makers is concerned, given the importance of these in translating drivers into realised changes in land use (van Vliet et al., 2015, Brown et al., 2016a, Brown et al., 2016b). The profit-based assumptions about decision-making used in CLIMSAVE models do not allow for a variety of effects that may occur in reality, from disjointed, protectionist responses to local or national changes in livelihoods, to migration, conflict or other societal disruptions (e.g. O'Brien et al., 2006, Black et al., 2011). Therefore, while these results do not depend upon an artificially fragmented conceptualisation of land system dynamics, they remain conditional upon aspects of model design that are not explored through our simulations or through comparisons with other models.

Nevertheless, it is clear that the challenges associated with climate change will be far larger in some parts of Europe than in others. While robustly productive or unproductive areas will have relatively little need or scope for adaptation, areas that are currently marginal may face complex and difficult choices, which do not realistically include the maintenance of existing land uses. In these places, the opportunities and processes of adaptation need to be carefully considered if the essential contributions of land use to human society are to be preserved.

Policy has an important role here, in smoothing transitions to more multi-functional management that may not otherwise arise without considerable disruption to livelihoods and environments. Policies that ease preferred changes in land use, rather than attempting to prevent change, are likely to be more successful in maintaining livelihoods, food supplies, and essential ecosystem services. Indeed, consideration of the full range of land-based services will be essential if synergies and trade-offs between them are to be appropriately managed, and it will also be necessary for policy-makers to consider a wide range of possible future conditions, as well as broad European and global contexts. In Europe, the extent of coherency between national policies will be important, and a decisive factor is likely to be the extent to which the future Common Agricultural Policy encourages the provision of societal goods such as climate mitigation, biodiversity preservation and livelihood diversification over economic production. In the absence of such support, reactive adaptations to the impacts of socio-economic and climatic changes are likely to be detrimental at local and European scales.

## 5 Conclusions

The CLIMSAVE IAP has been used to simulate the spatial distribution of four broad land use classes (intensive agriculture, extensive agriculture, very low intensity/unmanaged land and forest) across Europe in the 2050s under a broad range of climatic and socioeconomic conditions. Sixty climate scenarios spanned the equivalent of at least the 25th to 75th percentile range of changes in summer and winter precipitation and temperature change to 2065 within RCP2.8 to RCP8.5. These were combined with baseline socio-economics (climate change only) and four socioeconomic scenarios (climate and socioeconomic change) to understand the certainty in the direction of change of future land use allocation and the certainty in maintaining an unchanged proportion of land use classes into the future. Results suggested that climate change alone will lead to a contraction in the agricultural and forest area within Europe, particularly in southern Europe, which is partly offset by socioeconomic changes in both the demand for agricultural production, through changing food demand and net imports, and the efficiency of agricultural production. Whilst this modelling shows significant areas in northern and western Europe in which it is likely (> 66% probability) that current land use proportions will remain largely unchanged, the results reinforce current trends and previous integrated model findings of intensification of agriculture in northern and western Europe and extensification and abandonment in the Mediterranean region. These changes are driven by decreases in the relative profitability of the agricultural sector in southern Europe, owing to decreased productivity as a consequence of increased heat and drought stress and reduced irrigation water availability. The very low likelihood (< 33% probability) that current land use proportions in many parts of Europe will remain unchanged in the future suggests that policy should seek to promote and support the multifunctional role of agriculture and forests that recognises their contribution to the economic, environmental and economic functions of different European regions rather than focusing on supporting increased productivity as a route to agricultural and forestry viability.

## Figures and Tables

**Table 1:** Likelihood of baseline land cover proportions remaining unchanged (± 5%) across modelled grids under 2050s climate and socioeconomic change.

Likelihood scale	Probability	Climate change (% of grids)	Climate and socio-economic change (% of grids)
Virtually certain	99–100%	17	8
Very likely	90–99%	3	4
Likely	66–90%	8	12
About as likely as not	33–66%	11	16
Unlikely	10–33%	12	28
Very unlikely	1–10%	7	13
Exceptionally unlikely	0–1%	42	20

## Appendix A Supplementary data

Socio-economic drivers and additional model outputs. Image 1

## Footnotes

1 CLimate change Integrated Methodology for cross-Sectoral Adaptation and Vulnerability in Europe
