# Supplementary material for: Can we be certain about future land use change in Europe? A multi-scenario, integrated-assessment analysis
Source: Agric Syst. 2017 Feb;151:126–35. doi: 10.1016/j.agsy.2016.12.001 (PMC5268336; doi:10.1016/j.agsy.2016.12.001)
Supplement: Supplementary file 1 — Socio-economic drivers and additional model outputs. [file mmc1.docx]

**Supplementary Material**

**Table SM1** Europe-wide changes in selected socio-economic drivers under the four CLIMSAVE socio-economic scenarios for the 2050s (adapted from Harrison et al., 2015b)

| **Variable (% change from baseline)** | **Icarus** | **We are the World** | **Should I Say or Should I Go** | **Riders on the Storm** |
| --- | --- | --- | --- | --- |
| *SOCIAL DRIVERS:* | | | | |
| Population | -9 | 5 | 23 | 16 |
| Dietary preferences for meat | 10 | -21 | 0 | -9 |
| Water savings due to behavioural change | -30 | 45 | 11 | 52 |
| *TECHNOLOGICAL DRIVERS:* | | | | |
| Agricultural mechanisation | 10 | 44 | 5 | 77 |
| Water savings due to technological change | -35 | 29 | -60 | 45 |
| Agricultural yields | -9 | 15 | -3 | 26 |
| Irrigation efficiency | -9 | 26 | -21 | 58 |
| *ECONOMIC DRIVERS:* | | | | |
| GDP | 0 | 94 | -36 | 54 |
| Food imports | -6 | -13 | -13 | -13 |
| Bioenergy production | 6.7 | 1.8 | 1.8 | 6.7 |
| Oil price | 210 | 73 | 163 | 210 |

**Figure SM1 Robustness of the multi-scenario certainty in the direction of land use change (for change thresholds of 0.1 to 25% within a grid cell) for the 2050s for (left) climate change only and (right) climate and socioeconomic change**


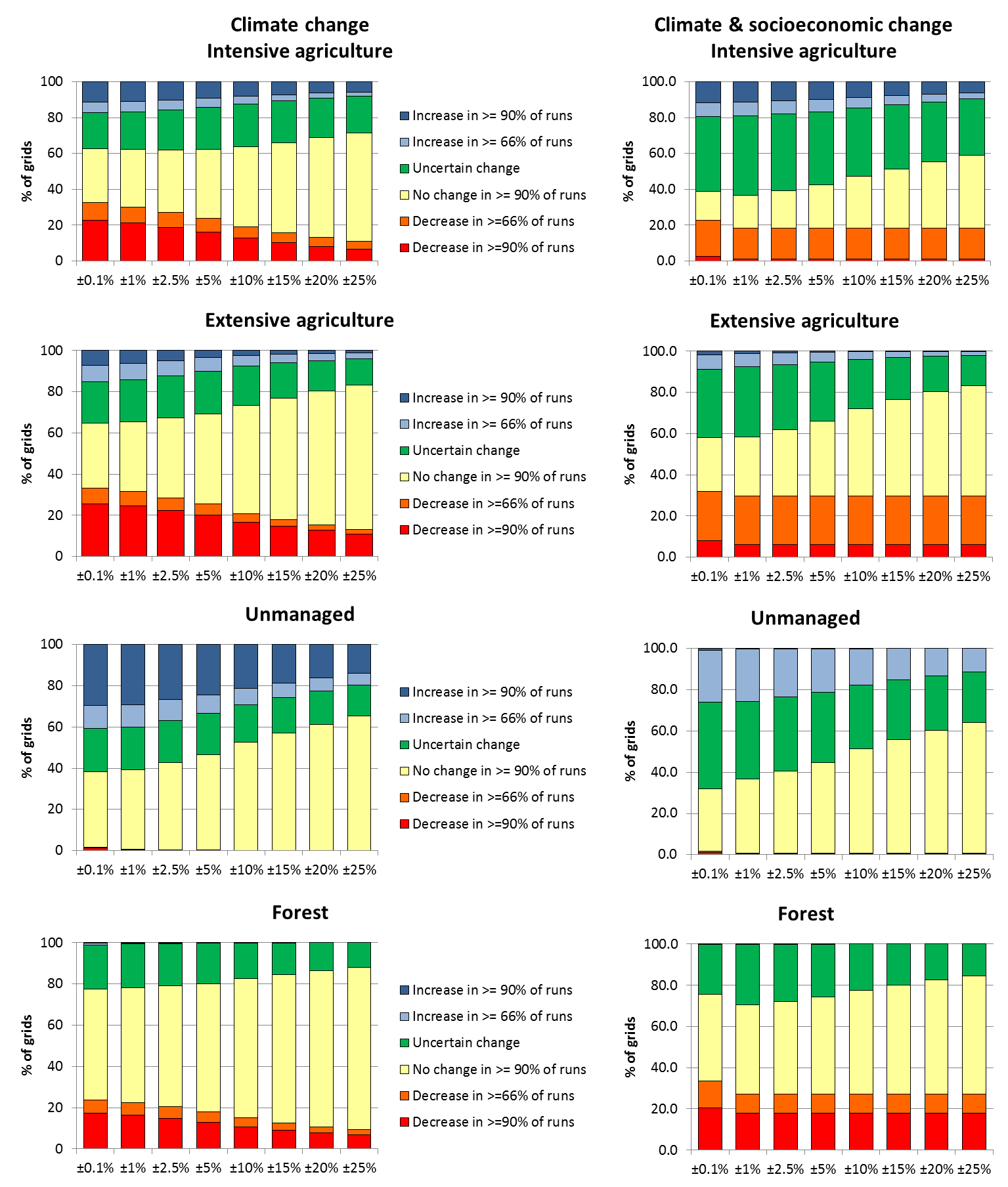


**Figure SM2 Comparison of the simulated baseline distribution of extensive agricultural land with the multi-scenario mean of the [60] simulations with climate change only and the [240] simulations with climate and socio-economic change for the 2050s**

**
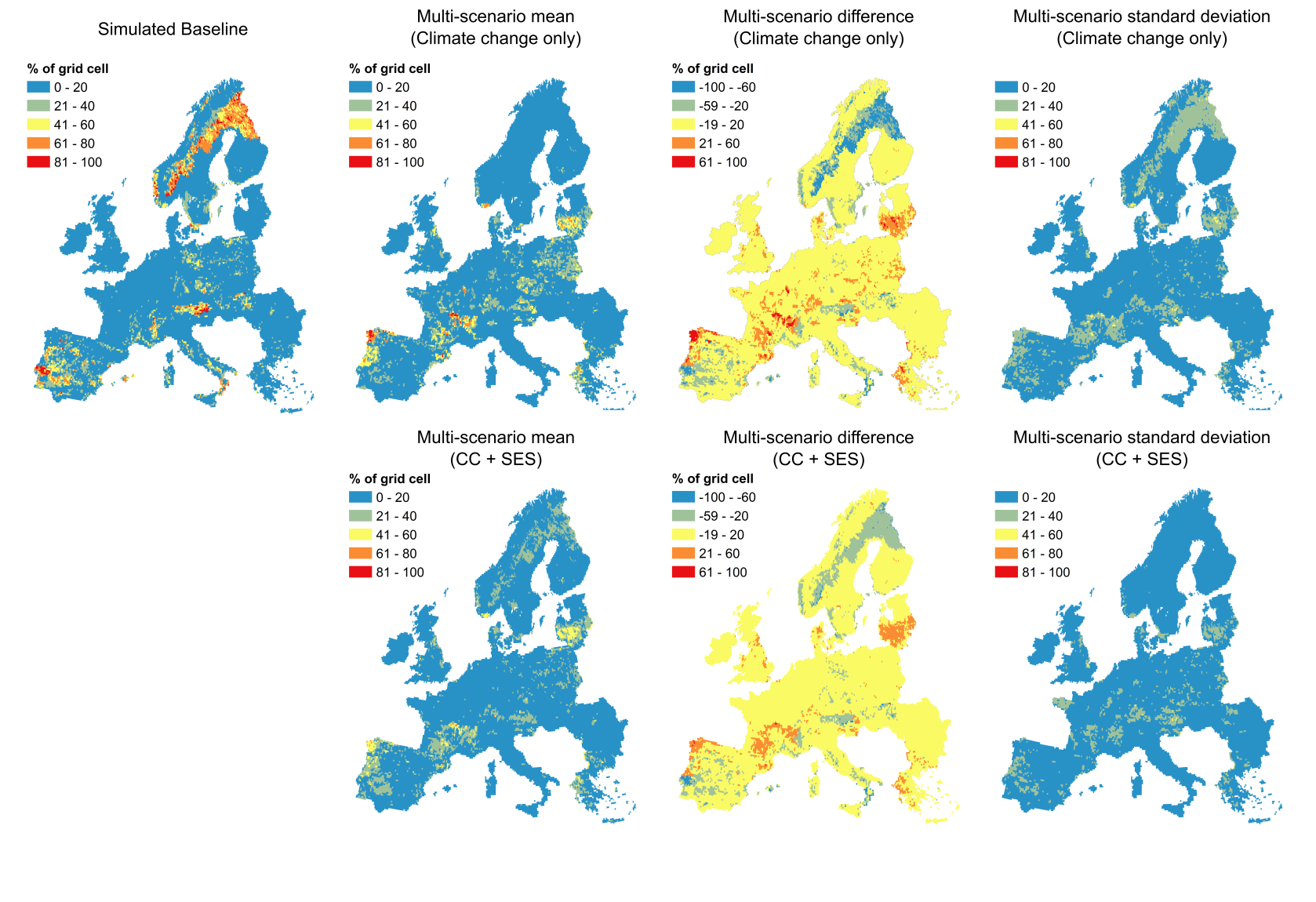
**

**Figure SM3 Comparison of the simulated baseline distribution of forest with the multi-scenario mean of the [60] simulations with climate change only and the [240] simulations with climate and socio-economic change for the 2050s**

**
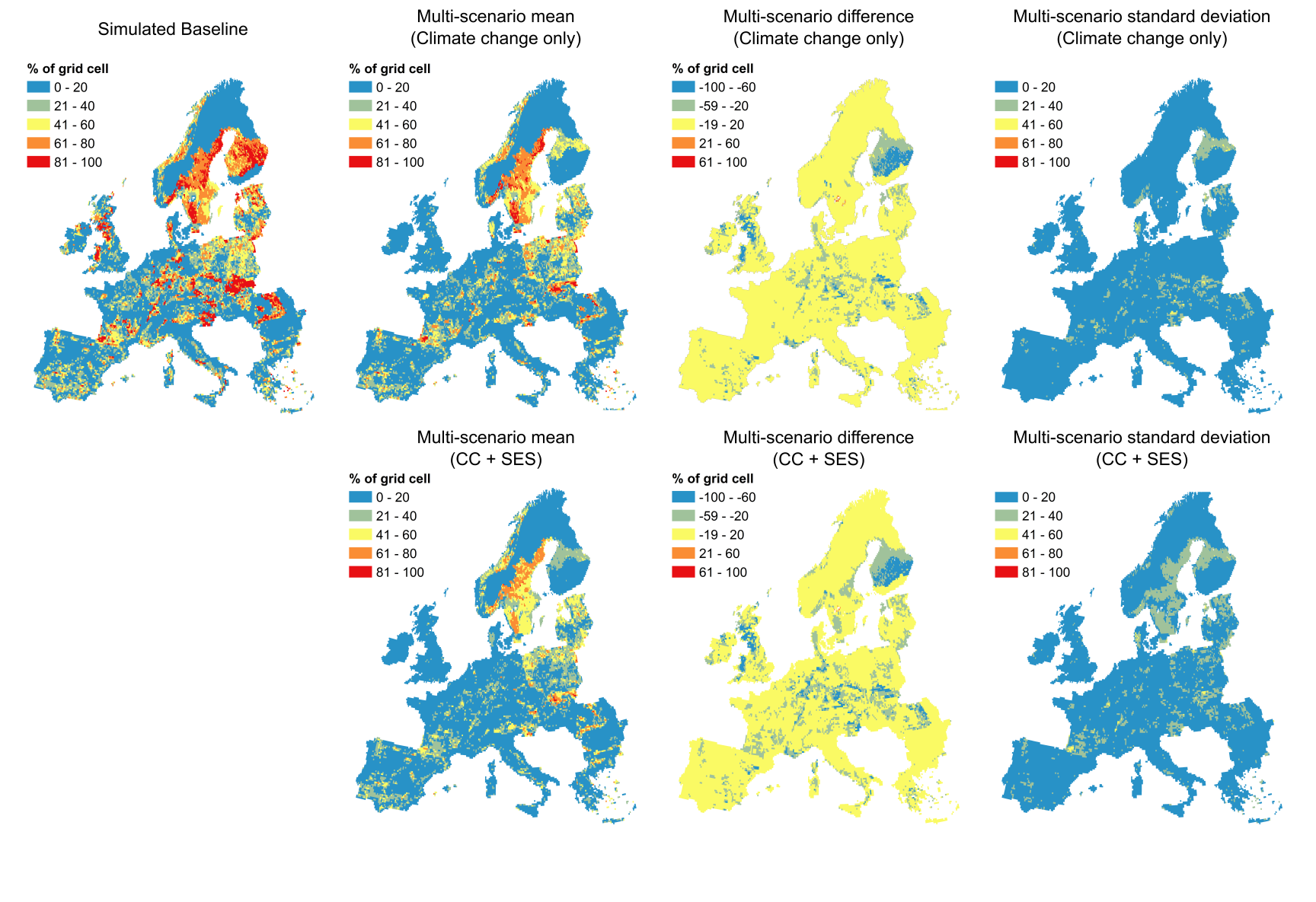
**

**Figure SM4 Comparison of the simulated baseline distribution of unmanaged land with the multi-scenario mean of the [60] simulations with climate change only and the [240] simulations with climate and socio-economic change for the 2050s**


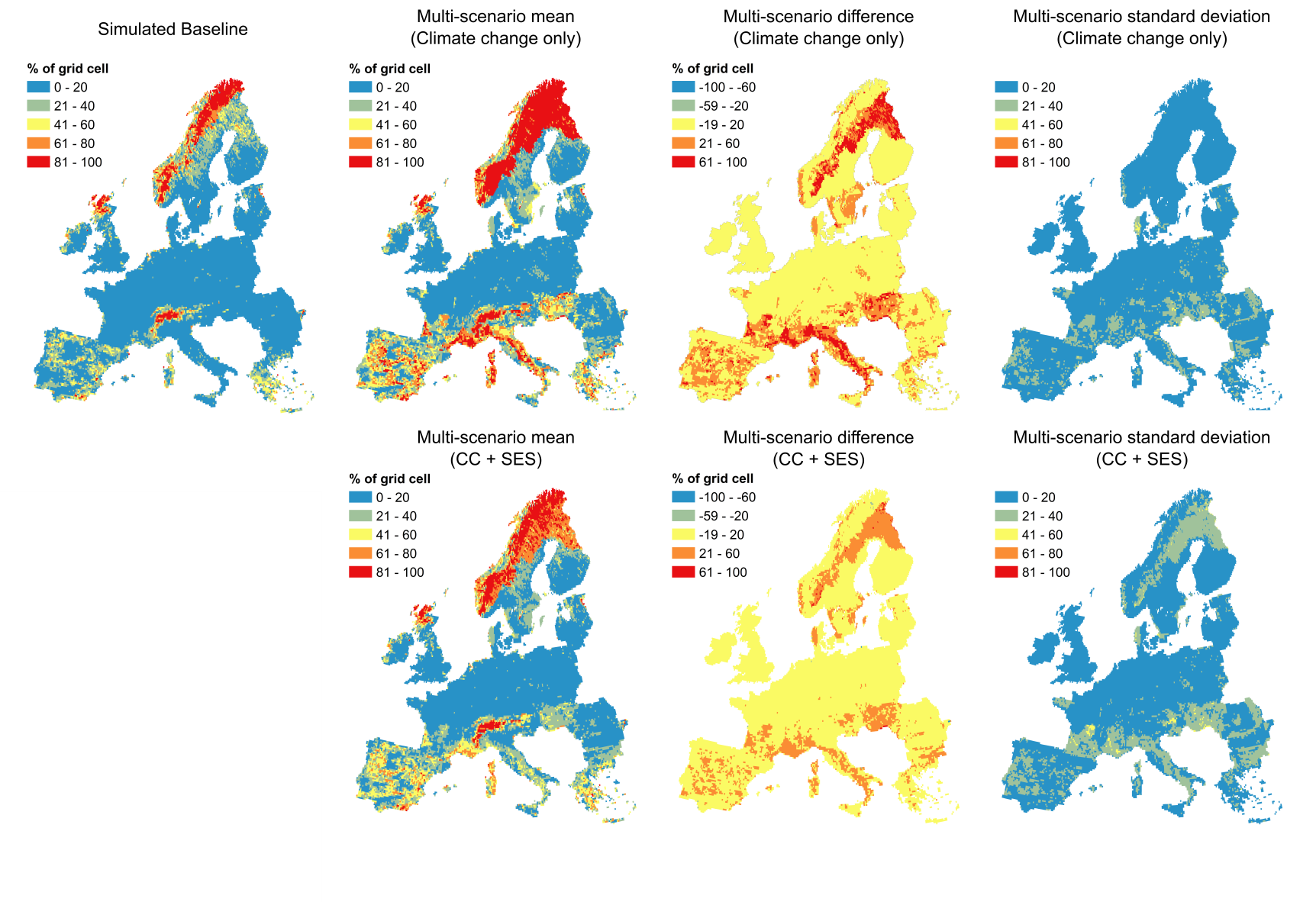


**Figure SM5 Multi-scenario certainty in the direction of land use change (of at least 5% within a grid cell) for the 2050s for (upper) climate change only and (lower) climate and socioeconomic change, expressed as percentage of grid cells**

**
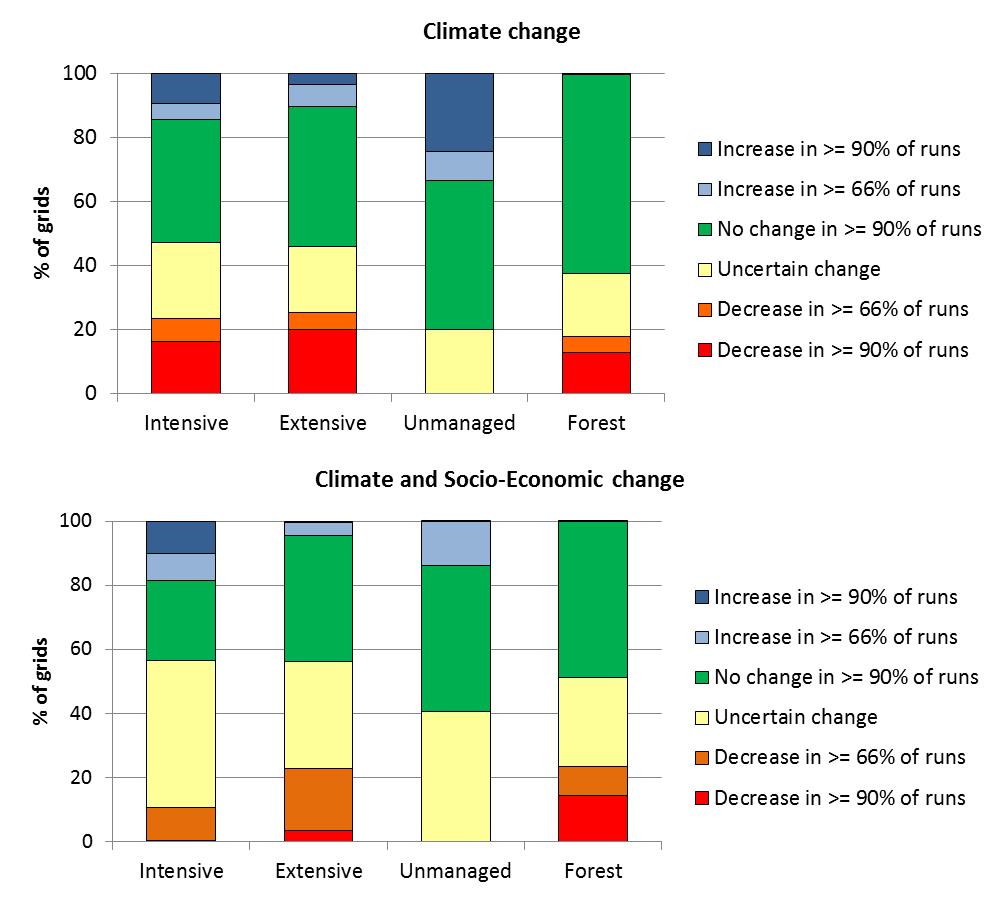
**
